# Hearing in Noise Test (HINT) in pediatric stuttering: a cross-sectional study of speech perception in noise performance in children who stutter

**DOI:** 10.1590/1516-3180.2026.3618.17092026

**Published:** 2026-11-13

**Authors:** Tugce Gul Caglar, Belde Culhaoglu, Seyra Erbek

**Affiliations:** I PhD. Audiologist, Assistant Professor, Department of Audiology, Faculty of Health Sciences, Lokman Hekim University, Ankara, Türkiye.; II PhD. Audiologist, Assistant Professor, Department of Audiology, Faculty of Health Sciences, Lokman Hekim University, Ankara, Türkiye.; III MD. Ear Nose and Throat Diseases, Professor, Department of Otolaryngology, Faculty of Medicine, Lokman Hekim University, Ankara, Türkiye.

**Keywords:** Stuttering, Speech Disorders, Hearing in Noise Test, Noise, Pediatrics, Fluency Disorder, Speech Perception, Hearing Performance, Children, Turkish HINT-C test

## Abstract

**BACKGROUND:**

Stuttering has a multifactorial, dynamic structure and is an involuntary fluency disorder characterized by disruptions in verbal expression. Stuttering is a common speech issue that affects people between the ages of 6 and 12.

**OBJECTIVES:**

The aim of this study was to compare speech perception in noise performance in children with developmental stuttering and their typically developing peers.

**DESIGN AND SETTING:**

A cross-sectional, observational study was conducted at Lokman Hekim University.

**METHODS:**

This study comprised 48 children in total, 24 of whom were 6 to 12 years old and complained of stuttering when they visited the Audiology, Hearing, and Balance Center; the remaining 24 children had no speech disorders. The children who participated in the study were administered the Hearing in Noise Test and underwent an audiological examination.

**RESULTS:**

The mean age of the 24 children with stuttering was 10.87 ± 3.67 years and that of the 24 healthy children was 12 ± 3.84 years. Children with normal hearing who did or did not stutter were included. A comparison of the results of the Hearing in Noise Test for children with fluency disorders and stuttered revealed that the stuttering group’s scores were lower, and this difference was identified as statistically significant (p < 0.001).

**CONCLUSION:**

Based on our findings, children who stutter experience greater difficulty with speech perception in noisy environments. This finding is important for the development of children aged 6 to 12 because difficulties in understanding speech in this developmental period negatively affect the social and academic lives of children with stuttering.

## INTRODUCTION

Individual differences in the developmental processes in children exist, and each child is at a different level of development. Differences and delays in a child’s developmental process affect their language and motor, cognitive, social, and daily lives. Language, which is a part of communication, has a significant impact on social development.^1^ Children with communication disorders may have difficulty grasping the meaning or use of language or expressing their thoughts or feelings through language. A direct proportional correlation exists between language development and communication.

Children with speech disorders experience difficulties in communication and in their social life. Stuttering, one of the speech disorders, is found in 1% of children worldwide.^2^ According to 10th revision of the International Classification of Diseases (ICD-10),^3^ stuttering is characterized by repetitions or prolongations of sounds, syllables, or words, or hesitations or pauses that disrupt the rhythm of speech.^4^ Stuttering is divided into two types, developmental and neurological, with developmental stuttering being more common.^5^ Currently, insufficient information is available on the frequency and prevalence of neurogenic stuttering.^6^ In developmental stuttering, stuttering can limit the speaker’s ability to participate in speech, leading to feelings of loneliness^4^ and a lack of self-confidence^7^; this may result in problems in interacting socially, which may be accompanied by social anxiety. Stuttering has a negative emotional, behavioral, and cognitive impact on the life of the speaker.^4^

Although studies involving the brain and behavior of stuttering have been conducted, no definite information on the causes of stuttering have been determined. Functional imaging methods were used in a recent study, demonstrating a relationship between stuttering and brain activity.^8^

When evaluating stuttering, generally the severity and psychological effects of stuttering are evaluated. These evaluations comprise subjective evaluation methods in which family participation, or the emotions of the individual with stuttering, are evaluated. Although some methods can be used to objectively evaluate stuttering, the evaluation results are subjective because of the mood changes or social anxiety of the person who stutters.

Based on previous literature, we hypothesized that children who stutter would demonstrate poorer speech-in-noise performance than typically developing children. In our study, we aimed to compare the results of the Hearing in Noise Test (HINT) between children with normal hearing who stutter and children with normal speech development.

## METHODS

This study was conducted at the Audiology, Hearing and Balance Center at Lokman Hekim University. A priori power analysis was performed using G*Power software to determine the required sample size. Based on the power analysis (power = 80%; α = 0.05; β = 0.20), a total of 48 children were included in the study, comprising 24 children diagnosed with stuttering and 24 age-matched children without any speech disorder (control group).

Ethical approval for the study was obtained from the Lokman Hekim University, Medical and Health Sciences Research Board (approval ID: 2023/155), as well as from the Lokman Hekim University, Human Ethics Committee. The parents or legal guardians of all participating children were informed about the study procedures, and written informed consent was obtained prior to participation. The study was designed as a cross-sectional case-control investigation.

### Participants

The inclusion criteria for the study were as follows:

(a) children diagnosed with stuttering,
(b) children currently receiving stuttering therapy, and
(c) children aged between 6 and 12.

Twenty-four children aged 6 to 12 who were diagnosed with stuttering and twenty-four children who had typical speech development participated in the study. All participants had normal hearing thresholds (0-15 dB HL) and were healthy, with no additional neurological, cognitive, or otologic problems.

All children underwent a comprehensive otolaryngological examination, including the collection of demographic data, a pure-tone audiometric evaluation, and administration of the Hearing in Noise Test for Children (HINT-C).

### Audiological assessment

Pure-tone audiometry and the HINT-C were conducted by a single experienced practitioner in a quiet test room. To facilitate comprehension and adaptation to the test procedure, speech reception thresholds were measured first in quiet and subsequently in noise settings.

### Pure-tone audiometry

Pure-tone audiometry was performed using an Interacoustics AC40 audiometer (Interacoustics, Middelfart, Denmark) following the otolaryngological examination. Air- and bone-conduction thresholds for both ears were measured separately across frequencies ranging from 125 to 8000 Hz using supra-aural headphones in a sound-treated booth. Only children with air-conduction thresholds of between 0 and 15 dB HL at all tested frequencies were included in the study.

### Hearing in Noise Test

The HINT is used to assess an individual’s ability to understand speech in noisy environments by requiring participants to repeat sentences that are presented under various listening conditions. The test includes digitally recorded sentences that can be administered in quiet and in the presence of competing noise; these sentences are administered simultaneously from two loudspeakers positioned at different angles. The HINT test is a computer-based assessment method that has been validated and shown to be reliable across different languages and age groups (pediatric and adult), and it is easy to administer.^9^

Cekic and Sennaroglu demonstrated the validity and reliability of the Turkish version of the HINT.^10^ The HINT is a computer-based assessment tool validated in multiple languages and is applicable to both pediatric and adult populations.

In this study, the HINT-C was used. The test was administered via the HINT-PV software by selecting the “Turkish-Child” language option and the “Fixed-Level HINT” protocol. During the pediatric assessment, children were instructed to repeat sentences presented from standardized sentence lists specifically designed for pediatric populations. Sentences were presented through two loudspeakers placed one meter apart, and each correctly repeated word was individually scored.^11^ ^,^ ^12^ ^,^ ^13^ The final scores were calculated as a percentage of the correctly repeated words.

In the experimental setup, one loudspeaker was positioned in front of the participant (Gfront: speech and noise at 0°), and the other was positioned to the left (Gleft: speech at 0° and noise at 90°). Separate recordings were obtained for each child.

### Statistical analysis

The IBM SPSS Statistics for Windows version 25.0 (IBM Corp., Armonk, New York) statistical program was used for the statistical analyses. Continuous variable statistical program numerical data are expressed as mean ± standard deviation. The numerical data were compared between the two groups using Student’s t-test and within each group using the paired t-test. A ρ value of less than 0.05 was accepted as statistically significant.

## RESULTS

Our study included a total of 48 children, 24 of whom were diagnosed with developmental stuttering and 24 of whom had normal speech. The children had a mean age of 11.43 ± 3.76 years. Of the children, 22 (45.8%) were girls and 26 (54.2%) were boys. The mean age of the children who stuttered was 10.87 ± 3.67 years, and that of the children who did not stutter was 12 ± 3.84 years. Statistically, no differences were observed between the two groups (p = 0.306). The stuttering group consisted of 7 female and 17 male children, whereas the control group comprised 15 female and 9 male children. When the two groups were compared in terms of age and gender, a statistically significant between-group difference was observed for gender, but not for age. After controlling for gender, the group effect on the Noise HINT-C scores remained statistically significant.

The results of the HINT-C test performed in quiet and noise environments were compared between the children with and without stuttering. In both groups, no significant difference in the HINT-C scores was observed when the test was performed in a quiet environment; however, a statistically significant difference was noted in the scores for tests performed in a noise environment (p < 0.001) **(** Table 1 **)**.

**Table 1:** Results of the Hearing in Noise Test conducted under quiet and noisy conditions

Variable	Children who stutter (Mean ± SD) n = 24	Controls (Mean ± SD) n = 24	p Value
Quiet HINT-C score (%)	100 ± 0	99,87 ± 0,61	0,323
Noise HINT-C score (%)	75,7 ± 11,53	95,63 ± 4,59	< 0.001*

* Independent samples t-test; significance level, p < 0.05. HINT-C, Hearing in Noise Test for Children.

## DISCUSSION

Stuttering is a speech disorder, and the underlying neurological mechanisms are not fully understood.^14^ Different theories have been proposed to explain stuttering, but according to the ASHA, it is an interruption of the flow of speech characterized by its occurrence in certain types of situations, and includes the repetition of sounds, syllables, words, consonant prolongations, and blockages. In this form of speech, the rate and rhythm of speech are affected and this may be accompanied by negative reactions to speech, avoidance behaviors, and physical tension.^15^ Developmental stuttering occurs in two different types, developmental and neurological stuttering; developmental stuttering is more common in early childhood. Acquired, or neurogenic, stuttering occurs after a neurological trauma in the developmental period or in adulthood and is less common. Stuttering that occurs in the developmental period negatively affects language development.^16^ Stuttering, one of the most common speech disorders, is frequently encountered in the developmental period; therefore, the aim of this study was to evaluate, in noise, the hearing of children who stutter.

Communicating in a noisy environment is difficult for children with speech and language disorders.^11^ Developmental, cognitive, and academic success are negatively affected by communication disorders. Stuttering limits participation in social situations, causes feelings of isolation and frustration, and has a holistic effect on the quality of life of people who stutter, leading to difficulties in education and employment and increasing the likelihood of mental health problems.^4^

The incidence of stuttering is four times higher in male individuals than in female.^2^ When we analyzed the data of the children participating in our study, we noted that the stuttering group consisted of more boys than girls (F:M, 7:17). Similarly, in their study involving children with stuttering, Zablotsky et al.^17^ reported that the incidence of stuttering was higher boys than in girls in the United States. Briley et al.^18^ evaluated the relationship between stuttering, depression, and suicidal ideation according to gender differences: The results of their study revealed that the number of male participants who stuttered was higher than that of female participants who stuttered. Consequently, stuttering is considered to be more common in male children.

Socially and psychologically, noise has a negative effect on life, and the intelligibility of speech is reduced in different environments. Martin et al.^19^ investigated noise sensitivity in stuttering: Their study included 48 people, 24 with stuttering and 24 controls. The results of the study indicated that children who stuttered were affected by noise with amplified sidetone conditions. Lotfi et al.^20^ evaluated children aged 7 to 12 years with stuttering using the Gaps in Noise test; their results revealed that, in the stuttering group, individuals with stuttering with normal DP obtained low Gaps in Noise test scores. Although hearing in noise tests have been conducted using different noises, the speech comprehension scores were also low in this study.

The study by Koca et al.,^21^ in which the relationship between stuttering and auditory processing was examined, included 30 children with and 30 children without stuttering, aged 8 to 17. The Frequency Pattern Test, Duration Pattern Test, and Gaps in Noise test were administered to all participants to evaluate auditory processing. The study results revealed that, among children with and without stuttering, children with stuttering had lower test scores, and that this was due to an auditory processing disorder.^21^

A recent meta-analysis on the role of the temporal cortex in stuttering revealed that little activation was observed in the part of the superior temporal sulcus located just in front of the areas found to have sound-selective auditory representations.^22^

Several studies examining the relationship between stuttering and the central system have been reported in the literature; the findings of these studies suggest that the basal ganglia-thalamocortical circuit is the neural network connection related to stuttering. The basal ganglia are an important component of the neural network related to stuttering because they modulate the activity of the left motor and temporal cortices. Lu et al.^23^ reported that, in individuals who stuttered, significant activation differences in the basal ganglia-thalamocortical existed, particularly in the left frontal motor cortex, temporal cortex, and basal ganglia regions. The critical role of the basal ganglia in stuttering has recently been described in a comprehensive review.^24^

The treatment administered for stuttering involves a number of different approaches, including pitch modification, word pronunciation facilitation, speech rate reduction, choral speech, fluency modification-modeling mechanisms, metronome speech, and white noise. The effects of differences in therapies on the duration of recovery are still unknown.^6^ All the children included in our study had been undergoing individualized stuttering therapy for at least 1 year, but the effectiveness of the therapies was not evaluated in this study.

The basal ganglia play a crucial role in both motor control and in cognitive processes such as timing, attention, and sensorimotor integration.^25^ Understanding speech in noisy environments relies on auditory perception as well as on the ability to selectively attend to speech signals, process temporal information, and rapidly interpret auditory input. Basal ganglia dysfunction can impair these processes, making it more difficult to distinguish speech in the presence of background noise. In individuals who stutter, timing and processing deficits associated with basal ganglia abnormalities may further exacerbate difficulties in speech perception under noisy conditions. Therefore, clarifying the link between basal ganglia function and speech perception in noise would strengthen the neurological foundation of the study.

The findings of this study showed that the test scores of children with stuttering decreased in noisy environments. The test batteries used in this study are current and widely used in audiology. To the best of our knowledge, this is the first study to be listed in PubMed in which children with stuttering were evaluated using the HINT-C test.

The results suggest that objective and subjective tests evaluating central functions in people who stutter are important for understanding the physiopathology of stuttering. The findings indicate that informative seminars in the scope of awareness studies, as well as pre-service and in-service training for teachers with students who stutter should be organized. Making changes to the studying, listening, or social environments of children with stuttering is necessary. Implementing these suggestions will positively affect the academic lives of children with stuttering. We believe that the findings of our study contribute to the research on stuttering and noise.

## CONCLUSION

In the present study, we demonstrated that children with stuttering exhibit significantly reduced speech perception performance in noisy environments, highlighting an important and often overlooked aspect of stuttering that extends beyond speech fluency. These findings support the growing body of evidence that suggests that stuttering is not solely a motor speech disorder, but that it also involves deficits in central auditory processing and sensorimotor integration. The observed difficulties in hearing in noise may be associated with dysfunctions in the neural networks involving the basal ganglia and temporal cortex, which play a critical role in timing, attention, and auditory-motor coordination. From a clinical perspective, these results emphasize the importance of incorporating hearing-in-noise assessments into the routine evaluation of children who stutter. Early identification of such deficits may guide more comprehensive and individualized intervention strategies, including environmental modifications and multidisciplinary approaches in educational settings. Ultimately, a better understanding of speech perception in noise may contribute to improving the communication outcomes, academic performance, and quality of life of children with stuttering.

## Data Availability

The data that support the findings of this study are available from the corresponding author upon reasonable request. The data are not publicly available due to ethical and privacy considerations.
